# Low-intensity transcranial focused ultrasound of the amygdala modulates neural activation during emotion processing

**DOI:** 10.3389/fnimg.2025.1580623

**Published:** 2025-05-30

**Authors:** Kathryn C. Jenkins, Katherine Koning, Arman Mehzad, John LaRocco, Jagan Jimmy, Shiane Toleson, Kevin Reeves, Stephanie M. Gorka, K. Luan Phan

**Affiliations:** ^1^Institute for Behavioral Medicine Research, The Ohio State University, Columbus, OH, United States; ^2^Neuroscience Graduate Program, The Ohio State University, Columbus, OH, United States; ^3^Department of Psychiatry and Behavioral Health, The Ohio State University Wexner Medical Center, Columbus, OH, United States

**Keywords:** low intensity focused ultrasound, neuromodulation, fMRI, EFAT, task-based fMRI

## Abstract

**Introduction:**

Low-intensity focused ultrasound (LIFU) is a form of neuromodulation that offers increased depth of penetrance and improved spatial resolution over other non-invasive techniques, allowing for modulation of otherwise inaccessible subcortical structures that are implicated in neuropsychiatric pathologies. The amygdala is a target of great interest due to its involvement in numerous psychiatric conditions. While prior works have found that LIFU sonication of the amygdala can alter resting-state neural activation, only a few studies have investigated whether LIFU can selectively modulate the amygdala during task-based fMRI.

**Methods:**

We aimed to address these gaps in literature in a cohort of 10 healthy individuals. We utilized the well-validated Emotional Face Assessment Task (EFAT), which is designed to robustly engage the amygdala. We selected the fusiform gyrus and the thalamus as our non-target regional comparison measures due to their roles in facial and emotional processing. In succession, participants completed a pre-LIFU baseline fMRI, received 10-min of LIFU neuromodulation, and then repeated the baseline fMRI. To test our hypothesis, we conducted paired-samples t-tests assessing changes in amygdala, fusiform gyrus, and thalamic activation from pre to post scan.

**Results:**

We found that there was a significant decrease in left (*t*(9) = 2.286; *p* = 0.024) and right (*t*(9) = 2.240; *p* = 0.026) amygdala activation from pre-to-post sonication.

**Discussion:**

Meanwhile, there were no differences in activation of the left or right fusiform gyrus or thalamus. Our results indicate that LIFU of the amygdala acutely dampens amygdala reactivity during active socio-emotional processing.

## 1 Introduction

Mental health issues are highly prevalent, with nearly 1 in 5 adults suffering from a diagnosable mental health disorder (NIMH, 2021). Although there are numerous evidence-based treatments for a wide range of neuropsychiatric disorders, many patients do not respond to existing interventions and/or report unwanted treatment side-effects which can lead to premature discontinuation of treatment (Smith-Apeldoorn et al., 2019). Neuromodulation, a technique which leverages external electrical, chemical, or mechanical stimulation to modify activity of the central or peripheral nervous system, is being increasingly explored as an alternative to existing treatment approaches for neuropsychiatric pathologies (Krames et al., 2009). Neuromodulation techniques may be broadly divided into two categories, invasive or non-invasive. Invasive techniques like deep brain stimulation (DBS) are highly efficacious but require implantation of intracranial electrodes, introducing undesirable risks (Kenney et al., 2007). Non-invasive approaches present less risk, but can be limited by depth of penetrance, poor spatial resolution, or insufficient neural target engagement (Hoy and Fitzgerald, 2010; Romanella et al., 2020). This has prompted a search for neuromodulation techniques in the goldilocks zone of limited risk and high target engagement that strike a balance of safety and effective target engagement.

Low intensity focused ultrasound (LIFU) is an emerging neuromodulation technique which may address this gap in treatment. The stimulation technique leverages ultrasound, a mechanical wave in the range of >20 kHz, to traverse skull and dura with minimal power loss, and target tissues of interest in the deep brain (Bystritsky et al., 2011). LIFU provides improved depth of penetrance over other non-invasive approaches but does not require surgical placement (Fini and Tyler, 2017; Kubanek, 2018; Sanguinetti et al., 2020). The effects of sonication depend on the parameters, including frequency and duty cycle of stimulation. At high intensity (>200 W/cm^2^), ultrasound causes permanent lesions via thermal ablation (Moosa et al., 2019), and at lower intensity (<100 W/cm^2^) ultrasound alters neural activity without causing ablation (Spivak et al., 2022a). In both animal and human models, low frequency sonication has been shown to reversibly alter neuronal activity (Tufail et al., 2010; Kim et al., 2014; Lee et al., 2016a; Dallapiazza et al., 2017; Downs et al., 2016; Lee et al., 2016b; Lee et al., 2016c; Legon et al., 2018). Therefore, LIFU provides promise as a novel neuromodulation technique to alter neural activity of deep brain structures.

The amygdala is a neural region of high interest that has been previously difficult to target. The amygdala is involved in a diverse array of functions including emotion processing (Šimić et al., 2021; Rolls, 2023; Wood et al., 2014). In particular, the amygdala plays an integral role in valence and salience detection and hyperactivity of the amygdala in response to aversive socio-emotional stimuli is a hallmark of internalizing disorders (Armony et al., 2005; Wright et al., 2006; Feldker et al., 2016; Morey et al., 2012). Given its role in identifying and mediating response to threatening stimuli, it is unsurprising that the amygdala is highly implicated in the etiology of several psychiatric disorders including depression, anxiety, post-traumatic stress disorder, and psychotic spectrum disorders, including schizophrenia and bipolar disorder (Nikolenko et al., 2020; Siehl et al., 2022; Ho et al., 2019). In addition, studies have shown that treatment-related decreases in amygdala activation during emotion perception tasks correlate with greater reduction in psychiatric symptoms (Gorka et al., 2019). Taken together, the amygdala plays a significant role in psychiatric disorders and treatment related reductions in activation are associated with meaningful changes in clinical outcomes. Therefore, this structure represents a promising target for non-invasive neuromodulation techniques.

Prior studies have repeatedly demonstrated that LIFU can modulate cortical and subcortical neural function. In several animal models, LIFU reversibly modulates neuronal activity (Tufail et al., 2010; Kim et al., 2014; Lee et al., 2016a; Dallapiazza et al., 2017; Downs et al., 2016) and non-human primate models reveal similar results in subcortical structures including the amygdala (Folloni et al., 2019). Resting-state fMRI (rs-FMRI) studies have shown that sonication increases perfusion and may increase or decrease BOLD activity and functional connectivity (FC) depending upon sonication parameters (Chou et al., 2024; Kuhn et al., 2023). Taken together, these works suggest that resting-state amygdala activity can be modulated by LIFU sonication and prompts further investigation of behavioral relevance of amygdala sonication using task-based fMRI paradigms.

Very few studies to date have investigated the pre- to post-task effects of LIFU sonication on amygdala activation. Chou & colleagues found that sonication decreased BOLD amygdala activation during a fear-inducing task. In addition, a recent study displayed that sonication of the amygdala enhanced acquisition of neutral emotional memories as well as enhanced fear recognition in faces (Doss et al., 2025). Task-based fMRI provides many benefits such as the ability to link brain activity to specific emotional processes through manipulation of task conditions and stimuli (see Stevens, 2016 for review). Importantly, task-based fMRI allows for engagement with clinically relevant processes. Many studies have shown that patients and controls differ greatly on neural activation during task-based emotional challenges (Etkin and Wager, 2007; Kujawa et al., 2016). Historically, much of this literature has focused on the use of social threat tasks. Hence, the current study aimed to investigate limbic reactivity in response to social signals of threat or distress immediately after treatment with targeted ultrasound. The Emotional Face Assessment Task (EFAT) is a validated probe of amygdala reactivity in response to social threat, e.g., fearful or angry faces (Bigos et al., 2008; Carre et al., 2014; Fonzo et al., 2015; Phan et al., 2008) and is utilized in the present study as a within subject pre-and post-measure of amygdala reactivity. While prior works have established the efficacy of resting-state neural activity modulation using LIFU sonication, this work is an important addition to the growing body of LIFU research that expands on our understanding of activation changes during functional engagement of the target structure. Confirming target engagement and demonstrating successful modulation of amygdala reactivity using LIFU neuromodulation is imperative to further establish the feasibility and efficacy of LIFU as a novel non-invasive intervention for therapeutic application.

The aims of the current study were to address these gaps in literature by directly assessing the effects of LIFU sonication on the functional engagement of the amygdala during a social threat task. We hypothesized that sonication of the target region would lead to localized suppression of engagement to aversive stimuli while sparing other non-target regions known to be implicated in facial processing.

## 2 Methods

### 2.1 Participants

Participants were recruited as a part of a single-arm open-label pilot study to investigate the effects of targeted LIFU sonication on the left amygdala with in-subject comparison of pre-post measures of amygdala reactivity. Participants were recruited from the central Ohio region using print advertising, online postings, and word-of-mouth referrals. Participants were required to be generally healthy and between the ages of 18–30 years old. Exclusionary criteria included any major active medical or neurological illness including history of epilepsy or seizures, current or prior history of psychological disorders, current psychotropic medication use, lifetime history of alcohol or substance use disorders, contraindications to MRI, and pregnancy (Sheehan et al., 1994). Written informed consent was obtained from all participants. During the consent process, participants were informed that the open-label protocol involved: (1) using low-intensity focused ultrasound (LIFU) to modulate brain activity, (2) targeting the left amygdala, which plays a role in emotion regulation, and (3) using MRI to measure the temporary effects of the ultrasound on brain activity. A total of 15 participants were enrolled. Four participants did not meet criteria for participation, and one was withdrawn due to non-compliance. Ten participants were included in the present study. All participants were monetarily compensated for their time. All study procedures were approved by The Ohio State University Institutional Review Board (2022H0087).

### 2.2 Procedures

#### 2.2.1 Clinician and self-administered assessments

By phone for follow-up and completed a side effect questionnaire which asked participants if they were currently experiencing or if they did experience any physical aftereffects from the procedure. No serious adverse events were reported by any participants.

#### 2.2.2 LIFU treatment

**Figure 1:**
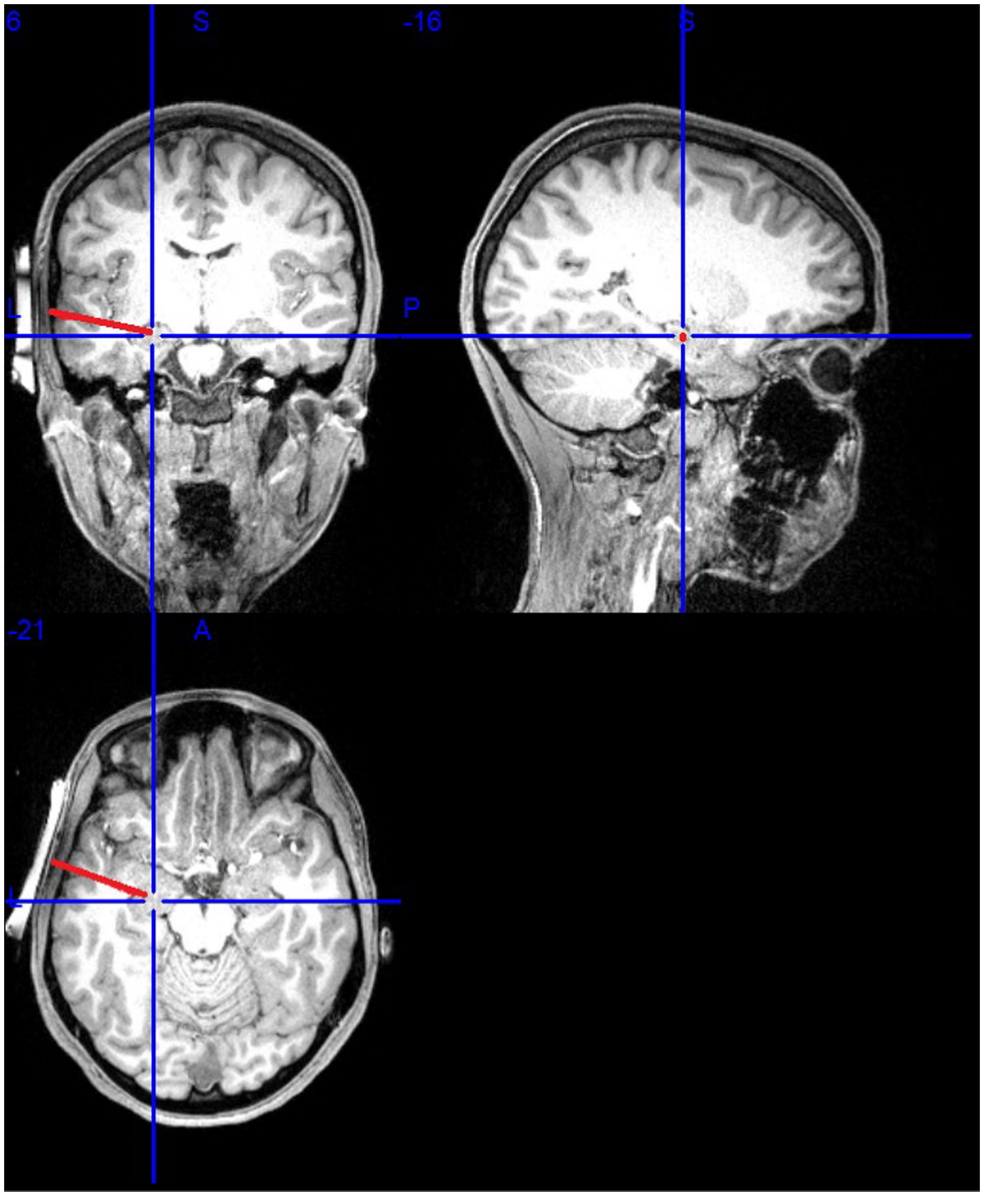
Illustration of the MRI-guided set-up.

Participants received sonication of the left amygdala (see Figure 1). The MR-guided sonication treatment was administered using the BrainSonix Pulsar 1002 LIFU pulsation system with a 65 mm transducer, which is compatible with MRI up to 3 T, allowing for precise, real-time target localization (Schafer et al., 2020). The transducer itself was independently tested and validated with a hydrophone, and LIFU parameters were simulated in Kranion software (Focused Ultrasound Foundation, Charlottesville, VA, USA). Acoustic and thermal modeling procedures, including assumptions regarding Gaussian beam profiles, skull attenuation, and one-dimensional k-Wave simulations, are detailed in Supplementary material. The transducer was positioned at the temporal window, using the participant’s temporal window as an anatomical landmark to ensure correct positioning. Even with manual positioning, the mounting system is designed to ensure correct transducer placement and minimize human error (Nouhoum et al., 2021). Confirmation occurred by examining the fiduciary markers on the transducer and structural scans. T1 weighted brain MRI scanning, rather than scout images, were then used to ensure proper placement of the LIFU transducer near the temporal window above the ear, where the skull is thinner and flatter to allow pulsed ultrasonic waves to travel through the skin and skull to reach subcortical targets. The LIFU transducer was secured using elastic straps and 5-degree angled gel pads to reduce dispersion and provide acoustic coupling (Fonzo et al., 2021). A second scan verified transducer positioning and sonication beam focus, with a target depth of 65 mm.

Once positioned, the LIFU transducer delivered 30-s trains of 650 KHz sonication at a pulse repetition frequency (prf) of 10 Hz with 30-s rest periods between sonication, for a total of 10 cycles over 10 min, with a spatial peak temporal average (ISPTA) of sonication was 720 mW/cm^2^ (Spivak et al., 2022b; Radjenovic et al., 2022). The stimulation was pulsed with a pulse width of 5 ms. The duty cycle was 5%. The mechanical index (MI) of the transducer was 1.7, below the FDA limit of 1.9. The estimated thermal dose was approximately 0.018 cumulative equivalent minutes at 43°C (CEM43°C) per sonication cycle, with a total dose of approximately 0.18 CEM43°C across the full session, remaining safely below the threshold associated with tissue necrosis (240 CEM43°C). These parameters are consistent with other studies which similarly aim to investigate amygdala modulation using LIFU stimulation (Fonzo et al., 2021; Philip and Arulpragasam, 2023).

#### 2.2.3 EFAT task

During each scan, participants completed two runs of a variant of the Emotional Face Assessment Task (EFAT) (Hariri et al., 2002). The EFAT, as detailed in prior works (Phan et al., 2008), reliably and robustly engages the amygdala target during fMRI (Hariri et al., 2002; Phan et al., 2013; Sripada et al., 2011; Labuschagne et al., 2010; Gorka et al., 2019). The task consisted of two types of matching blocks, matching faces and matching shapes. During matching faces, participants were presented with a trio of faces, one target face at the top of the screen and two below. Participants were instructed to identify which of the two faces below expressed the same emotion as the target face at the top of the screen. The target and congruent probe faces always displayed the same emotional expression, and a total of four different emotional expressions (fearful, angry, happy, and sad) were shown, while the incongruent face displayed a neutral expression. Each face had a unique identity, and an equal number of male and female faces were shown. All faces were extracted from a validated stimulus set (Gur et al., 2002). To allow limbic structures activated during match faces blocks to return to baseline and maintain the attention of participants, the match faces blocks were alternated with matching shapes blocks, during which participants were shown simple geometric shapes (i.e., triangles, rectangles, circles). As in matching faces blocks, participants were shown one target shape with two shapes below it and were instructed to identify the congruent shape. Each run of the task contained 24 experimental 20-s blocks: 12 blocks of matching faces, interleaved with 12 blocks of matching shapes. The 12 blocks of matching emotional faces contained three blocks of each of the four emotional types. Each experimental block contained four consecutive matching trials; each trial was 5-s long. The participant completed two runs of the task per scan where the block order was pseudorandomized.

#### 2.2.4 fMRI data collection

All scans were performed at the Ohio State University Center for Cognitive and Behavioral Brain Imaging (CCBBI), using a Siemens 3 T MAGNETOM Prisma MR scanner equipped with Total Imaging Matrix (TIM) system and a phase-array head coil to minimize signal loss and image distortion, and enable parallel imaging. Each session began with a T1-weighted anatomical scan acquired using a 3D MPRAGE sequence with the following parameters: repetition time (TR) = 2,400 ms, echo time (TE) = 2.24 ms, inversion time (TI) = 1,060 ms, flip angle = 8°, field of view (FOV) = 256 × 240 mm, matrix size = 320 × 300, slice thickness = 0.80 mm, 208 sagittal slices, and GRAPPA acceleration factor = 2. For each run of the EFAT task, 486 volumes were acquired using a multiband echo-planar imaging (EPI) sequence with the following parameters: TR = 1,000 ms, TE = 28.00 ms, flip angle = 60°, FOV = 240 × 216 mm, 45 axial slices, slice thickness = 3.00 mm (voxel size = 3 × 3 × 3 mm), and multiband acceleration factor = 3. To correct for geometric distortions in the EPI images, field maps were acquired using a dual-echo gradient-echo sequence with the following parameters: TR = 500 ms, TE1/TE2 = 5.17/7.63 ms, flip angle = 60°, FOV = 240 × 240 mm, 45 axial slices, and slice thickness = 3 mm. The acquired MRI data were stored and processed using storage and computing resources at Ohio Supercomputer Center (1987).

Functional imaging was preprocessed using the default options in fMRIPrep version 20.2.7 (Esteban et al., 2019). In brief, the steps included slice-time correction, distortion correction, head-motion correction, coregistration to subject’s own T1w image, and normalization to the Montreal Neurological Institute (MNI) template, specifically the MNI152NLin6Asym template. The preprocessed images were then smoothed with a 6 × 6 × 6 mm FWHM Gaussian smoothing kernel using the Statistical Parametric Mapping software (SPM12, Wellcome Department of Imaging Neuroscience, London, UK).

### 2.3 Region of interest analysis

A general linear model (GLM) was applied using SPM12 to the smoothed functional time series to model the task effects. The analysis focused on regional results due to the potential for false positives in a complex, whole-brain scenario. The task conditions were represented as boxcar functions and convolved with the hemodynamic response function to create the task condition regressor for the GLM. A high-pass filter with a 128-s cutoff, as implemented in SPM12, was applied. Six motion parameters (three translation and three rotation) estimated during head motion correction were included in the GLM as covariates of no interest to account for motion-related variability. Given the interest in amygdala function and anxiety (see Kim et al., 2011 for review), we created individual contrast maps for threat faces versus shapes (i.e., Fearful/Angry Faces > Shapes). We hypothesized that sonication of the amygdala target would be associated with acute reductions in amygdala activation to threat faces. Similar to other studies, we utilized a non-target regional comparison as an active control (Bergmann and Hartwigsen, 2021; Jung et al., 2016). It is important to note that the use of a regional control measure is not as rigorous as a placebo or sham condition. Because of this, we selected two regional control measures, the fusiform gyrus and the thalamus. We selected the fusiform gyrus due to its vital role in visual processes such as facial processing, object recognition, and reading (Weiner and Zilles, 2016). Multiple studies have shown that the fusiform gyrus is robustly activated while processing facial expressions during tasks like EFAT (Kawasaki et al., 2012; Monroe et al., 2013; Pujol et al., 2009). While the fusiform gyrus has been implicated in the perception of faces, literature suggests that it does not track the emotionality of facial expressions (Haxby et al., 2000). In addition, we selected the thalamus as a regional control measure due to its multifaceted role in emotional processing (Sun et al., 2015; Barson et al., 2020). Thus, the fusiform gyrus and the thalamus are prime candidates for our active control measures, allowing us to isolate activation strictly to the amygdala. Anatomical amygdala, fusiform gyrus, and thalamus masks from the AAL atlas were used to extract BOLD parameter estimates from threat faces > shapes from every subject pre-and post-sonication. Paired-samples t-tests assessing changes in amygdala, fusiform gyrus, and thalamic activation were then conducted. Consistent with our directional hypothesis, we reported the one-sided *p*-value associated with our paired-samples t-test. Family-wise error was used to adjust *p* values prior to statistical testing. There was no overall correction for multiple comparisons. Results with *p* < 0.05 were considered statistically significant.

A series of follow-up post-hoc analyses were conducted to examine differential effects in activation to each facial/emotional probe. We did not have any specific hypotheses regarding differential activation to emotional probes. Nonethless, we conducted a series of analyses which consisted of new contrasts for each emotional probe (angry faces > shapes, fear faces > shapes, and happy faces > shapes). In Supplementary material, we have included a whole-brain analysis to understand the spatial selectivity of LIFU sonication. In addition, we examined pre and post activation of specific amygdala subnuclei to our main contrast of interest (Angry/Fear> shapes).

## 3 Results

Sample characteristics are presented in Table 1. The mean age of participants was 22.91. The sample was predominantly white and was evenly split on biological sex. All participants were required to have no history of any psychological or neurological disorders. A post-hoc power analysis was conducted using G*Power 3.1.9.7 to determine the achieved power of a paired-samples t-test. The analysis was based on an observed effect size of *d* = 0.72 (left amygdala) and 0.71 (right amygdala), an alpha level of 0.05, and a sample size of *n* = 10. The results indicated that the test had 67.9% (left) to 66.3% (right) power (1 − *β* = 0.6788 and 0.6638, respectively) to detect the observed effect.

**Table 1:** Participant demographics and characteristics.

Demographics	
Age (years)	22.91 (3.14)
Sex (% female)	50%
Ethnicity (% Hispanic)	0%
Race	
White	50%
Black	30%
Asian	20%
American Indian or Alaskan Native	0%
Biracial, Other or Unknown	9%

### 3.1 Behavioral data

We first aimed to investigate whether any changes in our behavioral data occurred from pre- to post sonication. One participant was excluded from these analyses due to a technical issue that impeded collection of behavioral data. We found that there was no significant change to general task accuracy (*t*(8) = −1.153; *p* = 0.282). However, mean reaction time did decrease from pre- to post sonication (*t*(8) = 5.051; *p* = 0.001).

### 3.2 Pre-post amygdala activation

We assessed for changes in activation in our region of interest, the amygdala. Our results, presented in Table 2, revealed that there was a significant decrease in left and right amygdala activation from pre-to-post sonication.

**Table 2:** Experimental results from each EFAT contrast.

Contrast	ROI					
	Amygdala (L)	Amygdala (R)	Fusiform (L)	Fusiform (R)	Thalamus (L)	Thalamus (R)
Fearful/angry > shapes						
Pre-Tx	0.455	0.509	0.672	0.803	0.175	0.205
Post-Tx	0.318	0.306	0.604	0.711	0.113	0.126
*p*-value	0.024*	0.026*	0.214	0.163	0.189	0.169
*t*-value	2.286	2.24	0.828	1.037	0.929	1.012
Fearful > shapes						
Pre-Tx	0.536	0.643	0.697	0.838	0.229	0.259
Post-Tx	0.293	0.31	0.599	0.707	0.11	0.121
*p*-value	0.004*	0.037*	0.159	0.114	0.144	0.146
*t*-value	3.39	2.021	1.058	1.294	1.128	1.12
Angry > shapes						
Pre-Tx	0.373	0.376	0.646	0.769	0.122	0.151
Post-Tx	0.343	0.303	0.609	0.716	0.116	0.13
*p*-value	0.373	0.254	0.361	0.336	0.471	0.388
*t*-value	0.333	0.691	0.368	0.438	0.74	0.294
Happy > shapes						
Pre-Tx	0.373	0.466	0.622	0.743	−0.012	0.002
Post-Tx	0.226	0.31	0.539	0.711	0.009	0.051
*p*-value	0.181	0.169	0.27	0.411	0.433	0.319
*t*-value	0.962	1.014	0.636	0.231	−0.175	−0.486

*p < 0.05.

### 3.3 Pre-post regional control activation

**Figure 2:**
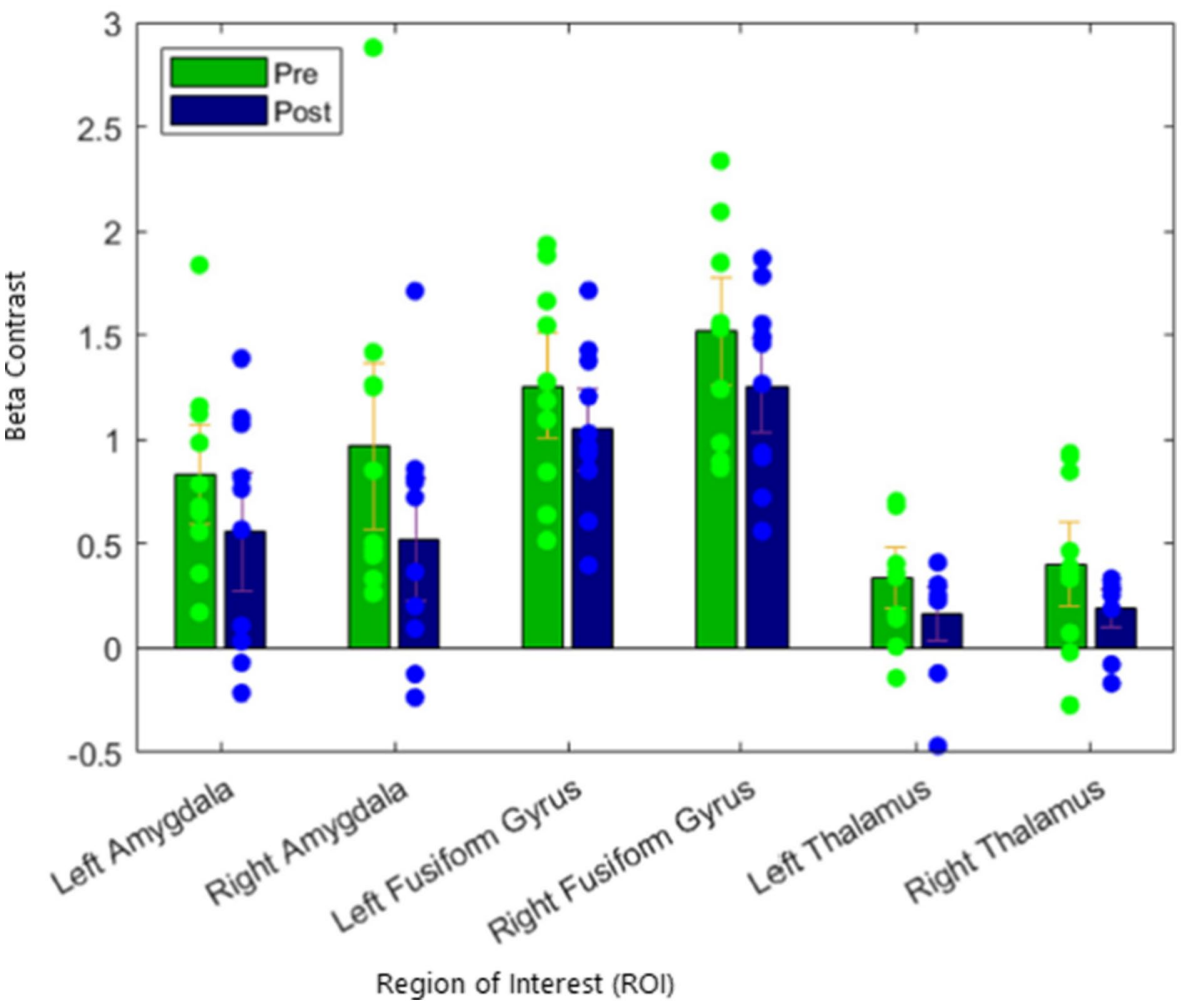
Reduction of activation of the amygdala target in fearful/angry > shapes contrast, with no significant suppression of regional control.

Next, we assessed for changes in activation in our regional control measures, the fusiform gyrus and the thalamus. We found that there were no differences in left or right fusiform gyrus activation from pre to post treatment. In addition, we did not find any differences in left or right thalamus activation. These results are presented in Table 2 and illustrated in Figure 2.

### 3.4 Post-hoc exploratory analyses

**Figure 3:**
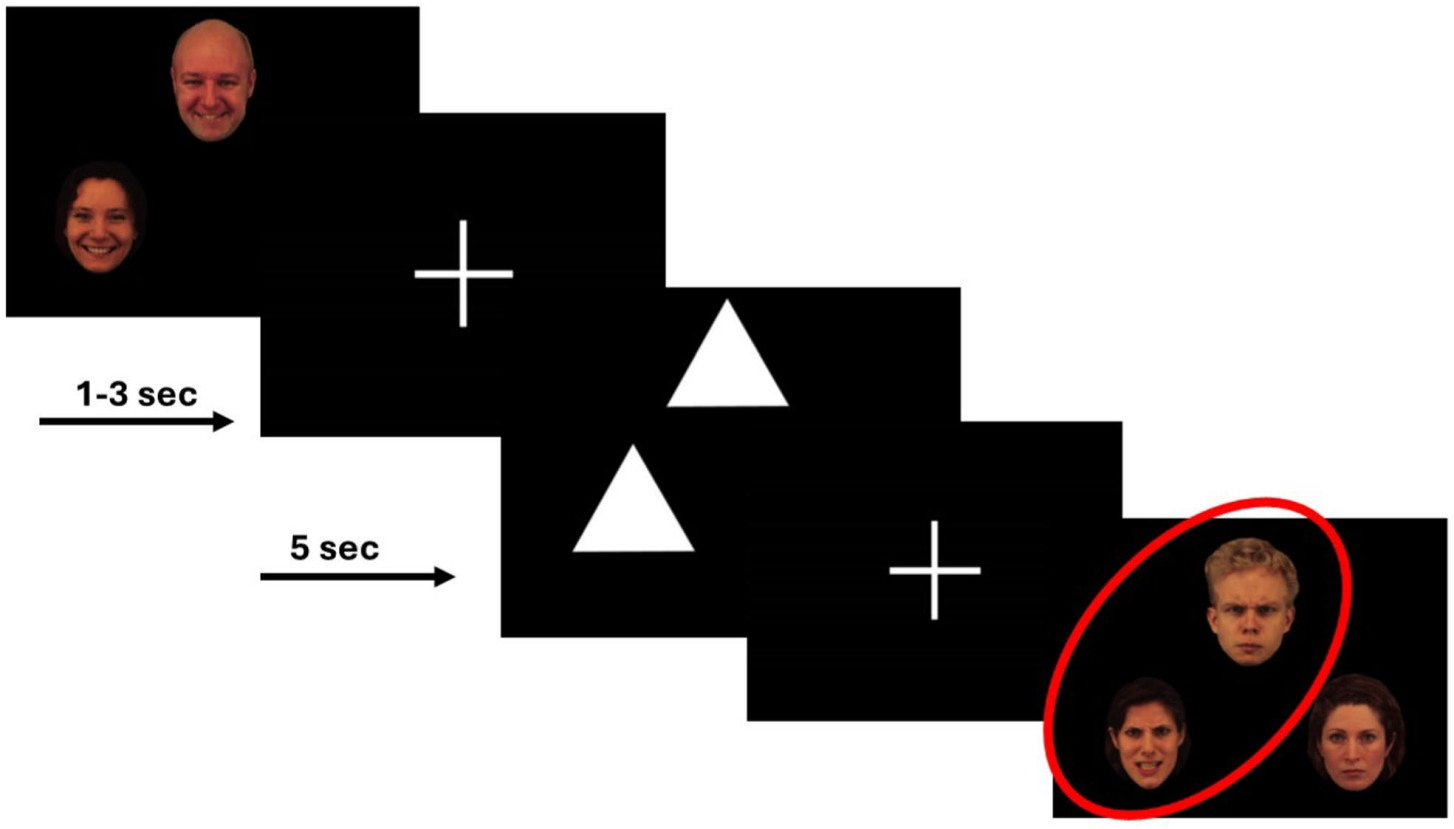
Example of the face-matching and shape-matching trials from the EFAT.

Additionally, we conducted a series of post-hoc analyses to explore differential activation effects to each facial/emotional probe. The original models were re-ran with new contrasts representing a distinct emotional probe > shapes. We found a significant change in right and left amygdala activation during fearful face trials, but not during angry or happy face trials. We did not observe any significant changes in activation of the fusiform gyrus or the thalamus to fearful, angry, or happy face trials (Figure 3).

## 4 Discussion

The aim of the current study was to assess the effects of LIFU sonication on amygdala activation utilizing task-based fMRI designed to directly probe amygdala function. Our results revealed that LIFU of the left amygdala was associated with a decrease in bilateral amygdala activation to threatening faces (relative to shapes). This effect is particularly robust in regard to fearful faces but was not observed in response to angry faces or happy faces. Notably, we did not observe significant changes in any of our contrasts from pre to post sonication in either of our regional control measures, the fusiform gyrus and the thalamus. These findings suggest that LIFU of the amygdala may modulate focal engagement of the target region during task-based, socio-emotional processing.

The amygdala plays a vital role in threat processing and hyperactivation in response to threat stimuli has been found to be related to psychopathology. The EFAT paradigm has been widely used to probe amygdala function and numerous studies have shown that fearful faces preferentially activate the amygdala (Hariri et al., 2002; Felmingham et al., 2010). This has also been observed in clinical populations, with studies displaying that individuals with anxiety and stress-related disorders exhibit increased amygdala activation to threatening faces relative to controls (Phan et al., 2013; Phan et al., 2008). It is for these reasons that we selected the amygdala as our primary sonication target. There have been many treatments and approaches geared toward attempting to modulate amygdala reactivity to threat stimuli. Approaches such as CBT and SSRI’s have been shown to successfully modulate reactivity of subcortical structures to threatening faces (Gorka et al., 2019; Fonzo et al., 2014). Importantly, not all individuals respond to existing therapies, nor do all patients evidence neural changes in the context of treatment. Neuromodulation techniques like LIFU are poised to fill an important gap by offering a potentially effective and selective tool for brain-based modulation. Along these lines, several recent studies have suggested that LIFU holds promise for changing amygdala function and, in-turn, improving psychopathology. For example, Mahdavi et al. (2021) recently demonstrated that clinical anxiety scores improved following 8 weeks of sonication treatment to the amygdala. This work highlights the necessity of linking changes in amygdala function via LIFU to changes in functional outcomes such as symptoms and behaviors. Though the present study was conducted in a population of healthy individuals, it nonetheless provides valuable insight into the feasibility of efficacy of LIFU as a potential non-invasive treatment for modulating amygdala activity in a clinical sample.

Task-based fMRI provides the unique ability to explore neural functional engagement while participating in tasks that elicit emotional states (Stevens, 2016). Very few studies to date have investigated if LIFU can modulate functional engagement of target structures during task-based fMRI. One notable study by Chou et al. (2024) utilized LIFU to target the left amygdala during a threat of shock task where participants received a mildly aversive shock at unpredictable intervals. They found that amygdala activation during the threat task decreased from pre to post session following LIFU stimulation and that this change was associated with a decrease in self-reported anxiety symptoms. The current study coincides with these findings by demonstrating that LIFU can modulate amygdala activation during a socio-emotional probe. Both tasks are objective threat measures, yet they capture different constructs of threat reactivity. A threat of shock task where the stimulus is unpredictable in its timing and duration elicits threat anticipation (Schmitz and Grillon, 2012). In contrast, exposure to angry and fearful faces elicits acute threat processing (NIMH, 2011). Regardless of the threat probe, there is now evidence that LIFU can robustly inhibit amygdala activation. In addition, our study revealed that the focal target (i.e., left amygdala) and its contralateral homolog showed suppressed engagement. This finding is consistent with previous studies that have displayed that sonication can modulate BOLD response in a target region and its associated networks (Kuhn et al., 2023). Taken together, our finding expands the current literature by demonstrating that LIFU of the left amygdala ameliorates amygdala reactivity to threatening faces while sparing changes in non-target regions.

Given that the current project was an open-label, one arm study, there was no sham condition included. We therefore utilized regional control measures to explore the specificity of our amygdala findings. The fusiform gyrus was selected as one of our control measures due to its critical role in higher-level visual processing, such as facial processing, object recognition, and reading (Weiner and Zilles, 2016). The fusiform gyrus has repeatedly been shown to be robustly involved in processing facial expressions during EFAT and other related paradigms (Kawasaki et al., 2012; Monroe et al., 2013; Pujol et al., 2009). On top of being a vital sensory relay center, the thalamus plays a key role in emotional processing (See Ward, 2013 for review). The thalamus is comprised of various nuclei, many of which contribute to modulating emotional processing and behavior (Sun et al., 2015; Barson et al., 2020). Our results revealed that there were no significant changes in activation in the fusiform gyrus or the thalamus from pre-to post treatment. This finding strengthens our results displaying that LIFU can selectively modulate functional engagement of a target structure and is not attributable to task-based habituation of structures involved in EFAT.

The current study had multiple strengths including the use of a well-validated socio-emotion processing task. The study also had several limitations. First, the study had a limited sample size and this did indeed limit the statistical power of our analyses; future studies should attempt to replicate these findings in a larger sample. In addition, our study focused on healthy individuals and therefore lacked a pathological participant population. Further research is required to understand the effects of LIFU in a clinical sample. Participants also displayed a decrease in their mean reaction time from pre to post sonication. This is likely due to habituation and is attributable to repeat administration of the task. While acoustic and thermal modeling confirmed expected pressure distribution and negligible heating at the sonication target (see Supplementary material for full details), full three-dimensional acoustic simulations incorporating individualized skull geometry and heterogeneity were not performed. Future work could incorporate subject-specific modeling to more precisely account for skull-induced beam distortion and focal shift. Lastly, the current study lacked a sonication sham condition. Given the statistical approach and limited sample size of the present project, we cannot be certain that an effect on the fusiform gyrus or thalamus was not present. Further investigation with an active control condition is required to link changes in amygdala to changes in self-report or other functional outcomes.

In conclusion, the current study provides evidence that LIFU sonication can suppress functional activation of regions involved in social threat processing. LIFU applied to healthy subjects resulted in suppressed recruitment of the amygdala target, during a task-based paradigm designed to robustly elicit activation. These results indicate that LIFU sonication can successfully move the amygdala target during the EFAT.

## Data Availability

The raw data supporting the conclusions of this article will be made available by the authors without undue reservation.
