# Supplementary material for: Low-intensity transcranial focused ultrasound of the amygdala modulates neural activation during emotion processing
Source: Front Neuroimaging. 2025 May 30;4:1580623. doi: 10.3389/fnimg.2025.1580623 (PMC12162705; doi:10.3389/fnimg.2025.1580623)
Supplement: Supplementary file 1 [file Data_Sheet_1.docx]

**Supplemental Material**

**Amygdala Subnuclei Analyses**

To further explore the impact of LIFU sonication on the amygdala, we examined pre-to-post activation of the basolateral, centromedial, and superficial amygdala subnuclei during threat faces (angry/fear) > shapes. Anatomical amygdala subnuclei masks from the CIT168 atlas were used to extract BOLD parameter estimates from every subject pre- and post-sonication. Paired-samples t-tests assessing changes in subgroup activation were then conducted. Our results revealed a significant decrease in the right (t(9)= -2.666, p= 0.012) not the left (t(9)=[ -1.753], p= 0.056) centromedial subregion. We also found that the left superficial subregion displayed a significant decrease in activation (L t(9)=[ -2.059], p= 0.0347). This was not observed in the right superficial subregion (t(9)=[ -1.061], p= 0.158). We found no significant changes in activation of the basolateral subregion in response to threat faces (L t(9)=[ -1.174], p= 0.135; R t(9)=[ -0.989], p= 0.174).

**Whole-Brain Analyses**

To test our hypotheses, we used a region-of-interest (ROI) approach. However, whole-brain analyses allow us to explore the impact of LIFU sonication beyond our targets and thus, we conducted a whole-brain, paired samples t-test using SPM 12. There were no significant changes at the whole-brain level using family-wise-error (FWE), p < .05 with at least 20 contiguous clusters as the significance threshold.

**Acoustic and Thermal Modelling Method**

Acoustic modeling was performed to estimate pressure distribution, temperature rise, and volumetric heating during LIFU sonication. Analytical modeling was based on a Gaussian beam profile generated by a circular aperture transducer with a focal distance of 65 mm (Ghoshal et al., 2011; Nahirnyak et al., 2007). Thermal effects were estimated using the Pennes bioheat transfer equation, assuming perfect energy absorption, no active perfusion, and no thermal diffusion cooling.

Skull attenuation was modeled at 2 dB/mm across and assumed 6 mm temporal bone thickness, resulting in an approximate 12 dB loss across the skull. In prior studies, temperature and pressure have been correlated with Gaussian beam intensity under similar assumptions, omitting perfusion cooling (Ghoshal et al., 2011; Nahirnyak et al., 2007).

Trajectory planning assumed sonication through the left temporal window targeting the left amygdala. The near-field region included the temporal lobe cortex and underlying white matter, with the full-width half-maximum (FWHM) region encompassing adjacent hippocampal structures. In the event of off-target misalignment, thalamic structures could also be affected; however, the Gaussian beam profile ensures rapid pressure and intensity falloff away from the focal zone, mitigating off-target effects.

Additionally, a one-dimensional acoustic simulation was performed using the k-Wave MATLAB toolbox (Treeby and Cox, 2010). The simulation modeled ultrasound propagation along the sonication axis through homogeneous tissue, using the same transducer and skull attenuation parameters.

**Acoustic and Thermal Modelling Results**

Analytical modeling predicted that the initial peak rarefactional pressure generated at the transducer surface was approximately 712 kPa. Following propagation through the skull, the pressure near the surface was estimated at approximately 178 kPa. Pressure increased along the beam profile, reaching approximately 444 kPa at the full-width half-maximum (FWHM) region and peaking at approximately 628 kPa at the focal depth of 65 mm.

Thermal modeling using the Pennes bioheat equation predicted that the maximum temperature rise at the focal point during a 30-second sonication cycle would be less than 0.1°C. Under worst-case assumptions of perfect energy absorption and no perfusion cooling, surface heating could theoretically reach up to approximately 0.45°C. Even modeling higher attenuation values for the skin and skull showed that the temperature increase remained negligible at the focal point, supporting the thermal safety of the protocol.

Additionally, a one-dimensional (1D) acoustic simulation was conducted using the k-Wave MATLAB toolbox, modeling ultrasound propagation through homogeneous tissue under the same parameters. The 1D simulation confirmed a peak focal pressure of approximately 0.58 MPa and verified a negligible thermal rise, with a maximum simulated temperature increase of less than 0.01°C. The results from both analytical modeling and numerical simulation were consistent, providing strong support for the expected acoustic and thermal behavior during sonication.

**Safety Assessment**

Thermal dose was assessed using cumulative equivalent minutes at 43°C (CEM43°C), applying standard thermal dose calculation methods (Dewhirst et al., 2003). Based on the predicted heating rates, the estimated thermal dose per 30-second sonication cycle was approximately 0.018 CEM43°C, and the total cumulative thermal dose over 10 cycles was approximately 0.18 CEM43°C. These values are well below the established threshold of 240 CEM43°C associated with thermal tissue damage, further supporting the safety of the applied sonication protocol. The estimated heating effects were comparable to those reported in low-intensity ultrasound imaging studies and were substantially lower than those associated with thermal ablation protocols (Chen et al., 2023; Ghoshal et al., 2011; Nahirnyak et al., 2007).

Mechanical Index (MI) was calculated as the peak rarefactional pressure (Pr.3) after attenuation divided by the square root of the frequency, resulting in an MI of approximately 0.778. This value is substantially lower than the U.S. Food and Drug Administration (FDA) diagnostic ultrasound safety limit of 1.9, indicating that the sonication protocol operated within established mechanical safety parameters.

In addition, post-sonication T1-weighted structural MRI scans were reviewed for any acute structural changes and to ensure tissue integrity. Participants were contacted via telephone approximately 24 hours after their session to complete a side effects questionnaire. The questionnaire assessed if they are currently experiencing or if they did experience any physical aftereffects from the procedure. No serious adverse events or delayed complications were reported by any participants.

**Discussion**

Our results revealed a significant decrease in activation from pre-to post sonication in the right centromedial and left superficial amygdala subregions in response to threat faces (>shapes). We observed no changes in activation to the basolateral amygdala subregion. Recognized as the main output hub of the amygdala, the centromedial subregion has extensive connections with the brainstem and hypothalamus (LeDoux., 2007). It receives input from the basolateral subregion, which is involved in appraising and ascribing valence to stimuli (Correia & Goossens, 2016). Through its extensive output connections, the centromedial subregion plays an important role in modulating behavioral and autonomic responses during a threat response. In this way, the centromedial subregion can directly drive defensive behaviors such as increased heart rate, pupil dilation, freezing responses, and hypervigilance (Mucarello & Penzo, 2022). Conversely, the superficial amygdala subregion is exclusively activated by facial expressions compared to non-social stimuli, suggesting that it plays a role in extracting social value from environmental cues (Goossens et al., 2009) We found that LIFU sonication can non-selectively decrease activation of these two key amygdala subnuclei. It is important to note that our ultrasound parameters were not designed to target specific subnuclei. Therefore, these post-hoc analyses are exploratory in nature and require further replication.

**References**

Chen, M., Peng, C., Wu, H., Huang, C.C., Kim, T., Traylor, Z., Muller, M., Chhatbar, P.Y., Nam, C.S., Feng, W. and Jiang, X., 2023. Numerical and experimental evaluation of low‐intensity transcranial focused ultrasound wave propagation using human skulls for brain neuromodulation. Medical Physics, 50(1), pp.38-49.

Correia, S. S., & Goosens, K. A. (2016). Input-specific contributions to valence processing in the amygdala. *Learning & Memory*, *23*(10), 534-543.

Dewhirst, M. W., Viglianti, B. L., Lora-Michiels, M., Hanson, M., & Hoopes, P. J. (2003). Basic principles of thermal dosimetry and thermal thresholds for tissue damage from hyperthermia. International Journal of Hyperthermia, 19(3), 267–294.

Ghoshal, G., Luchies, A. C., Blue, J. P., & Oelze, M. L. (2011). Temperature dependent ultrasonic characterization of biological media. The Journal of the Acoustical Society of America, 130(4), 2203-2211.

Goossens, L., Kukolja, J., Onur, O. A., Fink, G. R., Maier, W., Griez, E., ... & Hurlemann, R. (2009). Selective processing of social stimuli in the superficial amygdala. *Human brain mapping*, *30*(10), 3332-3338.

LeDoux, J. (2007). The amygdala. *Current biology*, *17*(20), R868-R874.

Moscarello, J. M., & Penzo, M. A. (2022). The central nucleus of the amygdala and the construction of defensive modes across the threat-imminence continuum. *Nature Neuroscience*, *25*(8), 999-1008.

Nahirnyak, V., Mast, T. D., & Holland, C. K. (2007). Ultrasound-induced thermal elevation in clotted blood and cranial bone. Ultrasound in medicine & biology, 33(8), 1285-1295.

Treeby, B.E., & Cox, B.T. (2010). k-Wave: MATLAB toolbox for the simulation and reconstruction of photoacoustic wave fields. *Journal of Biomedical Optics*, 15(2), 021314.
